# Long axial field-of-view PET imaging of intraarterial 2-[^18^F]FDG injection

**DOI:** 10.1007/s00259-024-06898-1

**Published:** 2024-09-12

**Authors:** Korbinian Krieger, Carola Maria Bregenzer, Luis Weissenrieder, Axel Rominger, Thomas Pyka

**Affiliations:** 1 Department of Nuclear Medicine, Inselspital, University Hospital Bern, University of Bern, Freiburgstrasse 18, CH-3010 Bern, Switzerland; 2 Center for Radiopharmaceutical Sciences, Department for Chemistry and Applied Biosciences, ETH Zürich, Zurich, Switzerland; 3 Cantonal Hospital Aarau, Aarau, Aargau Switzerland

The unintentional intraarterial (i.a.) injection of 2-[^18^F]FDG is a rare adverse event during PET/CT, resulting in excess activity in the extremity downstream of the puncture site known as *glove phenomenon* or *hot forearm sign* [1–5]. I.a. injections are distinct from extravasations as in latter cases, radioactivity in the subcutaneous tissue drains slowly via the lymphatic system, whereas i.a.-administered activity is swiftly carried into systemic circulation [6]. Excess nuclide observed after i.a. injection is the consequence of the “first pass”, the brief passing of the admixture of concentrated radiotracer and arterial blood through he capillaries of the extremity. In practice both cases are readily differentiated by visualization of lymphatic drainage and circumscript activity in subcutaneous tissue [7], both of which are absent following i.a. administration. SUV measurements are compromised by extravasation [8], but no data exists for i.a. administration. A case of i.a. 2-[^18^F]FDG administration in a 75-year old male undergoing PET/CT 60 min post injection for follow-up of pulmonary metastatic melanoma allowed us to longitudinally compare image quality. Eight point five per cent of injected activity were found in the affected forearm, compatible with previous reports suggesting approximately ten per cent first-pass extraction of 2-[^18^F]FDG in muscle [4]. Lesional uptake was not compared as the patient achieved complete remission on programmed cell death protein 1 inhibitors. As 2-[^18^F]FDG biokinetics are variable and deviations in SUV of ten to twenty per cent are common in routine scans [9], the deviation induced by i.a. injection is unlikely to be of grave consequence.
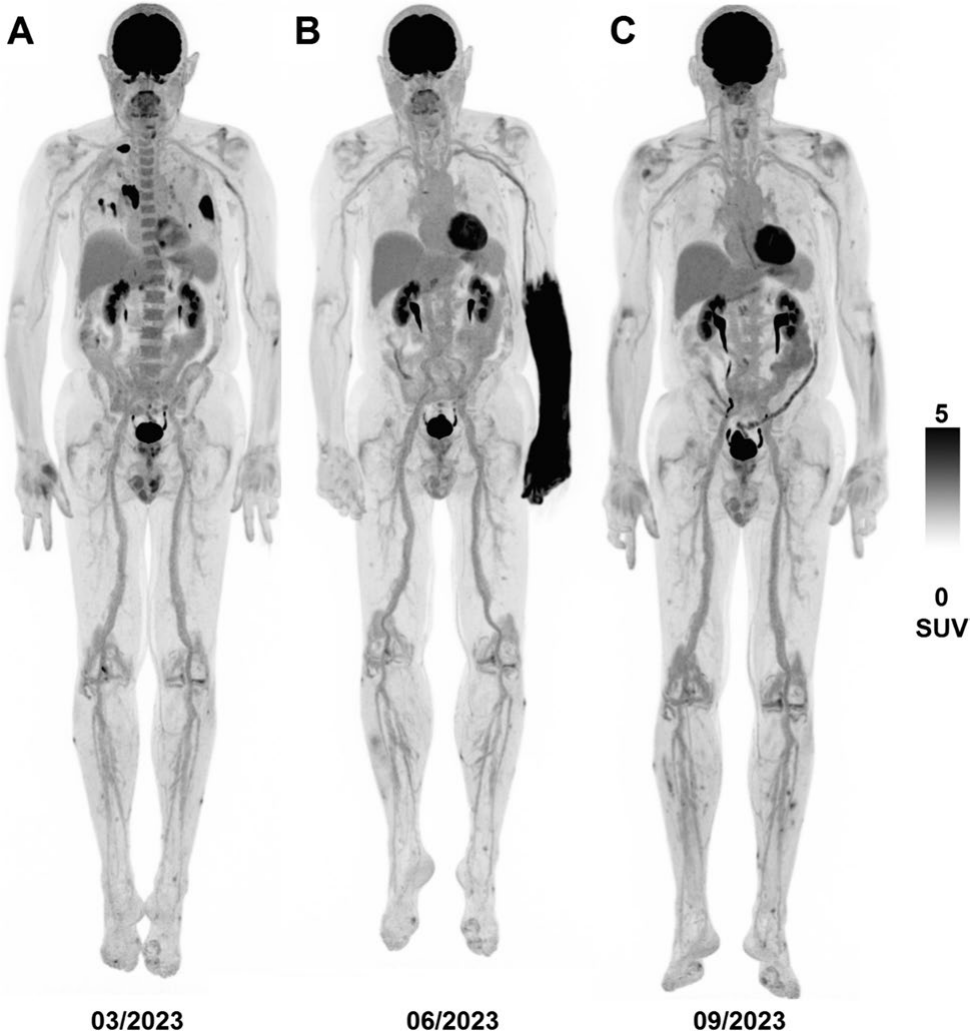


## Data Availability

Original data is available from the authors upon reasonable request, subject to applicable Swiss privacy and data protection law.
